# Explore Transplant at Home: a randomized control trial of an educational intervention to increase transplant knowledge for Black and White socioeconomically disadvantaged dialysis patients

**DOI:** 10.1186/s12882-015-0143-0

**Published:** 2015-08-28

**Authors:** Amy D. Waterman, Anna-Michelle M. McSorley, John D. Peipert, Christina J. Goalby, Leanne J. Peace, Patricia A. Lutz, Jessica L. Thein

**Affiliations:** Division of Nephrology, David Geffen School of Medicine at University of California, Los Angeles, 10940 Wilshire Blvd, Suite 1223, Los Angeles, CA 90024 USA; Division of General Medical Sciences, Washington University School of Medicine, Campus Box 8005, 660 S. Euclid Ave., St. Louis, MO 63110 USA; Missouri Kidney Program, University of Missouri, Columbia, AP Green Building, Suite 111, 201 Business Loop-70 W, Columbia, MO 65211 USA

**Keywords:** Kidney transplantation, Living donor, Racial disparities, African-Americans, Patient education, Health knowledge/attitudes, Transtheoretical model of behavioral change, Stages of Change

## Abstract

**Background:**

Compared to others, dialysis patients who are socioeconomically disadvantaged or Black are less likely to receive education about deceased donor kidney transplant (DDKT) and living donor kidney transplant (LDKT) before they reach transplant centers, often due to limited availability of transplant education within dialysis centers. Since these patients are often less knowledgeable or ready to pursue transplant, educational content must be simplified, made culturally sensitive, and presented gradually across multiple sessions to increase learning and honor where they are in their decision-making about transplant. The *Explore Transplant at Home* (*ETH*) program was developed to help patients learn more about DDKT and LDKT at home, with and without telephone conversations with an educator.

**Methods and Study Design:**

In this randomized controlled trial (RCT), 540 low-income Black and White dialysis patients with household incomes at or below 250 % of the federal poverty line, some of whom receive financial assistance from the Missouri Kidney Program, will be randomly assigned to one of three education conditions: (1) standard-of-care transplant education provided by the dialysis center, (2) patient-guided *ETH* (*ETH*-PG), and (3) health educator-guided *ETH* (*ETH*-EG). Patients in the standard-of-care condition will only receive education provided in their dialysis centers. Those in the two *ETH* conditions will receive four video and print modules delivered over an 8 month period by mail, with the option of receiving supplementary text messages weekly. In addition, patients in the *ETH*-EG condition will participate in multiple telephonic educational sessions with a health educator. Changes in transplant knowledge, decisional balance, self-efficacy, and informed decision making will be captured with surveys administered before and after the *ETH* education.

**Discussion:**

At the conclusion of this RCT, we will have determined whether an education program administered to socioeconomically disadvantaged dialysis patients, over several months directly in their homes, can help more individuals learn about the options of DDKT and LDKT. We also will be able to examine the efficacy of different educational delivery approaches to further understand whether the addition of a telephone educator is necessary for increasing transplant knowledge.

**Trial Registration:**

ClinicalTrials.gov, NCT02268682

## Background

In the United States there are approximately 637,000 patients with end-stage renal disease (ESRD) or kidney failure [1]. There are two options for ESRD patients to sustain life: dialysis or a kidney transplant from a deceased or living donor [1–4]. Kidney transplantation, especially living donor kidney transplant (LDKT), offers ESRD patients 6 to 16 additional years of life and improved quality-of-life compared to remaining on dialysis [1–3]. However, the majority of ESRD patients–450,000 as of 2013– still remain on dialysis. Although dialysis is life-saving, it only replaces 10-15 % of normal kidney function and can lead to cardiovascular disease, infection, and other complications [5, 6]. Additionally, the chance of a dialysis patient being alive after 5 years without a transplant is only 40 % [7]. Acknowledging its benefits over dialysis, the Centers for Medicare and Medicaid Services (CMS) has required that all dialysis patients receive transplant education and be informed of their option for transplant within 45 days of initiating dialysis.

In general ESRD patients who are Black and of lower socioeconomic status (SES) are significantly less likely to receive transplant education, pursue transplant evaluation, and receive deceased donor kidney transplants (DDKTs) and LDKTs [8, 9]. In fact, patients with kidney disease from low-SES neighborhoods experience higher mortality rates [10]. These patients may be more likely to have particular concerns about the costs of transplant medication [11], loss of disability benefits if they should receive a transplant [12], and limited access to adequate transportation to transplant appointments [13, 14]. Many low-SES patients may receive treatment at dialysis facilities with systemic barriers, including limited staff availability to provide education about transplant [15–17] or limited access to transplant education materials [18]. Without information about the risks and benefits of DDKT and LDKT, dialysis patients who do not present at a transplant center are often unable to make informed decisions about how best to treat their kidney disease [19, 20].

In light of these barriers, a 2014 American Society of Transplantation Consensus Conference recommended multiple ways to improve educational outreach for ESRD patients who have not yet presented at a transplant center [21]. These recommendations included repeating education multiple times and in settings prior to the transplant center, providing more culturally competent education at appropriate literacy levels, and using technology more effectively as an educational strategy. These approaches reflect a more general need to provide transplant education both consistently and gradually over time for patients who know less about it. Patients with significant barriers to learning about transplant also may benefit from support from a health educator, peer mentor, or social worker [22], including repeated discussions and more opportunities to ask questions about their transplant concerns.

Given the pressures on dialysis providers’ time [16] health insurance companies have incorporated the use of case managers or health educators within their organizations to distribute health information to patients via mail or over the telephone [23]. Some approaches that have been used to reach patients in clinical settings outside of dialysis centers involve mailing videos and print educational content at multiple time points to reach patients and their support networks when they are home [24], sending regular text messages to increase health knowledge and promote healthy behaviors [25, 26], and reinforcing educational content with a phone health educator [23]. These approaches have been shown to be effective in identifying patients with chronic illness early in the course of disease progression and intervening to increase knowledge of the best treatment options available, bridge the gaps in access to care, facilitate effective communication between the patient and providers, advocate on behalf of the patient, and plan for successful treatment outcomes in a high quality and cost-effective manner [27–29].

Applying these educational strategies to transplant education for the first time, we describe the protocol of a randomized control trial (RCT) with low-SES, Black and White dialysis patients comparing the efficacy of two versions of the *Explore Transplant at Home* (*ETH*) educational program with standard education being provided within dialysis centers. The three education conditions include: (1) standard-of- care education provided within dialysis centers; (2) a video-guided four-part *ETH* program delivered via mail, plus optional texts [*ETH* Patient-Guided (PG)]; and (3) a video-guided four-part *ETH* program with discussion facilitated by a transplant educator via telephone [*ETH* Educator-Guided (EG)]. We will compare whether these three educational approaches improve Black and White patients’ transplant knowledge, self-efficacy, and informed decision-making. We will also explore which DDKT and LDKT action steps patients commonly take during an 8 month period.

## Study Design and Methods

### Foundations of ETH

There is general consensus that interventions grounded in the best practices of behavioral change theories are more effective than those not based in theory [30]. Noting that many dialysis patients, especially racial minorities, are in early stages of decision-making to pursue transplant [8], there is critical need for an intervention that can meet patients where they are in this process and gradually increase knowledge, leading them towards making informed transplant choices. Dr. Waterman and her team previously designed and tested the *Explore Transplant* (*ET*) program [19], a program grounded in the Transtheoretical Model of Behavior Change (TTM) [31]. The TTM holds that not all patients are ready to begin taking actions toward health behavior change, and that patients’ decision-making is impacted by their level of motivation, their weighing of the Pros and Cons, and their self-efficacy [31]. It has been successfully used to understand the decision-making of patients considering whether to engage in over 50 health behaviors [32–37], including decision making about organ donation [38, 39]. Through a group RCT, *ET* delivered face-to-face with patients while they were undergoing dialysis by transplant educators was shown to increase patients’ knowledge, informed decision-making, and pursuit of transplant [19]. As a result, the *Explore Transplant* program won the 2009 National Association of Transplant Professionals (NATCO) Quality of Care Award.

### Design and Advantages of Explore Transplant at Home

The original *ET* trial included four video and print educational modules: Exploring Transplant, Kidney Recipients’ Transplant Experiences, Living Donors' Donation Experiences, and Deciding What to Do. These modules were reviewed in person while the patients were undergoing dialysis within a one month period. Recognizing the significant barriers that disadvantaged dialysis patients face in pursuing transplant, the *ETH* modules were redesigned to be delivered by mail to patients’ homes, with content delivered more gradually, over an 8 month period, and in smaller educational increments using supplementary texts, postcards and, in one condition, telephone support from an educator (Table 1). Both *ET* and *ETH* were developed to encourage informed decision-making through a comprehensive explanation of the benefits and risks of dialysis, DDKT and LDKT.

**Table 1:** Explore Transplant at Home mailed intervention materials

Module	Brochures	Fact Sheets	Postcards	Videos
1	Explore Transplant: A Guide for Family and Friends	Transplant or Dialysis Fact Sheet	Your Exploration of Kidney Transplant Begins at Home	Exploring Transplant
	Why People Donate Their Kidneys	Deceased or Living Donation Fact Sheet	Explore Transplant with Your Friends and Family	
	Why Kidney Patients Get Transplants		Learn How Life Can Improve After Transplant	
2	–	Recipient Evaluation, Surgery and Recovery Fact Sheet	Learn Something New About Receiving a Kidney	Kidney Recipients’ Transplant Experiences
		Possible Risks to Kidney Recipients Fact Sheet	Compare the Risks and Benefits of Transplants	
			Learn What Transplant Evaluation is Like	
3	–	Living Donor Evaluation, Surgery and Recovery Fact Sheet	Learn What it is Like to be a Living Donor	Living Donors’ Donation Experiences
		Possible Risks to Living Donors Fact Sheet	Learn Why People Want to Be Living Donors	
			Compare the Risks and Benefits of Living Donation	
4	Deciding What to Do	Kidney Disease Resources Fact Sheet	Weigh the Pros and Cons of All Your Options	Deciding What to Do
		Missouri Transplant Centers Fact Sheet	Consider Living Donation	
			Plan Your Next Steps	
Totals	4	8	12	4

A Medical Advisory Board including social workers, case managers, nephrologists, kidney patients, and experts in health education reviewed and approved all components of the *ETH* educational intervention to ensure its cultural sensitivity and that the needs of low-income and low health literacy groups were addressed.

### Missouri Kidney Program

**Fig. 1:**
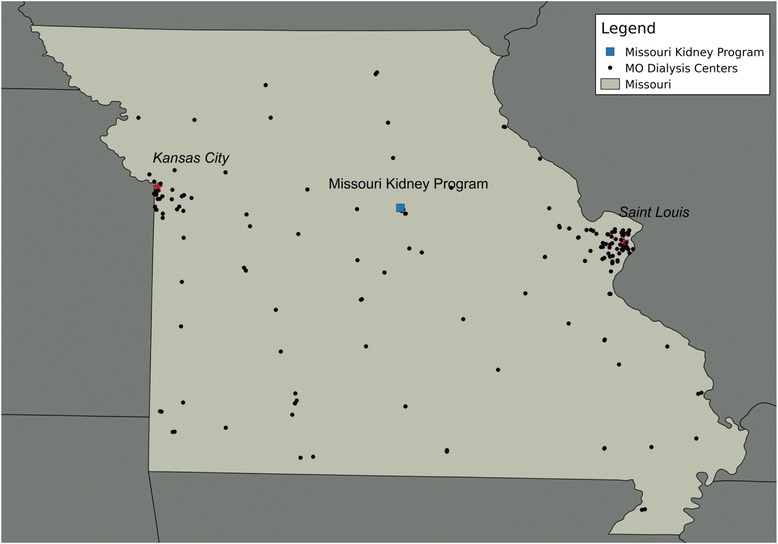
Intervention region: Missouri dialysis centers

This study features a partnership between Dr. Waterman’s transplant research team and the Missouri Kidney Program (MoKP) [40]. Established in 1968 to serve and educate Missouri’s citizens with chronic kidney disease (CKD), MoKP subsidizes the costs of dialysis and transplant medication for low-income ESRD patients in Missouri, effectively operating as an insurance company would with respect to their 1,200 patient member group. MoKP also has strong, statewide partnerships with over 160 dialysis centers across the state of Missouri (Fig. 1) and fosters the exchange of medical, technical and administrative information among programs and professionals who treat patients in these dialysis centers.

### ETH Educational Components

#### Printed Materials

Many of the printed materials, including brochures and factsheets, used as part of the *ETH* education program are part of the original *ET* Program [19]. Every 8 weeks, within the educational intervention period, patients in the *ETH*-PG and *ETH*-EG intervention conditions will receive four educational print and video modules by mail. Transplant education postcards will also be mailed to patients every two weeks following the mailing of each of the four modules, for a total of 12 postcards over the course of 8 months.

#### Videos

As part of their mailings, patients will receive four *ET* DVD videos, averaging 20 minutes in length, to review at home with people who help them make important health decisions. If patients indicate that they do not have a DVD player at home, one will be provided to them as part of the trial. The videos include the stories of 20 transplant recipients and living donors, and discuss the questions and fears they had before getting a transplant and why they became motivated to pursue transplant. The health professionals in the videos provide answers to common questions, including specifics about transplant evaluation, surgery and recovery processes involved with being a transplant recipient or a living donor. All videos are closed-captioned for the hearing impaired.

#### Text Messaging

Text messaging has become a popular form of communication [41] and a common way to receive updates and important information quickly and conveniently [42]. Healthcare providers are now utilizing these short messages to deliver reminders to patients about appointments, prescription medications, and health education [43, 44]. In the United States, 89 % of households have access to a cell phone [45]. In 2011, 73 % of those who owned cell phones used them to send and receive text messages [46]. Thus, to increase the ways we can reach patients with educational messages, the *ETH* program has incorporated the use of text messages that contain recommendations, quiz questions, and educational facts that complement the printed materials and videos during each module. (Table 2). Patients in both *ETH* intervention conditions will have the option of enrolling in an educational text messaging service designed to supplement the *ETH* education they are receiving in the mail. Patients in the *ETH*-PG and *ETH*-EG conditions will receive a minimum of 67 messages. Those who respond to interactive messages, such as quiz questions, could receive up to 80 messages over the intervention period. Patients who opt into the text messaging are provided $10 remuneration to cover the cost of standard text messaging rates. Text messaging services are provided through Songwhale LLC, [47] an institutional review board approved partner.

**Table 2:** Sample text messages

Text Message Type	Content
Recommendation	What does your family think about living donation as an option for you? Ask them! They know you the best and their opinions might help you decide.
Quiz	Can living donors who donate a kidney still have kids afterwards? Know the answer? Text back YES or NO.
Fact	Did you know? Nationally, most patients wait, on average, 4 years for a kidney from the deceased donor waiting list.

#### Transplant Educator

Patient support programs using health educators are increasingly being offered under health insurance plans [23] with staple components including patient assessment planning, facilitation, and advocacy [29, 48]. Though this intervention was not able to provide a comprehensive case management program, *ETH* has incorporated key tenets from case management models and created a telephone transplant educator whose goal is to guide patients through *ETH* to increase knowledge and informed decision-making (*ETH*-EG condition only). During a series of four calls, each lasting approximately 20 minutes, which will occur after each *ETH* module is mailed, the educator and patient will review the educational materials and discuss the risks and benefits of transplantation. The educator will also provide support by addressing patient concerns, problem-solving, and practicing empathetic listening. The transplant educator who delivers this intervention is a nephrology social worker who has had over 20 years of experience working with CKD patients. The educator has also received extensive training in the TTM and on how to administer *ETH*.

### Control Condition: Standard-of-Care

Patients randomized to the standard-of-care condition will not receive any educational materials from our program and will only participate in the survey portion of the investigation. The study team will conduct a phone survey to assess the actual educational practices occurring within each dialysis center by interviewing the dialysis providers who deliver transplant education. Research has shown that the most common educational practices in dialysis centers are recommending that patients learn more or be evaluated for transplant referring patients to an education program at a transplant center or kidney organization, and providing them with brochures one time [18]. Dialysis providers will be asked to continue their current practices throughout the study period without change. While Control patients will be free to ask additional questions or solicit more information from their dialysis educators at any point during the study period, no additional educational interventions will be delivered.

### RCT Overview

**Fig. 2:**
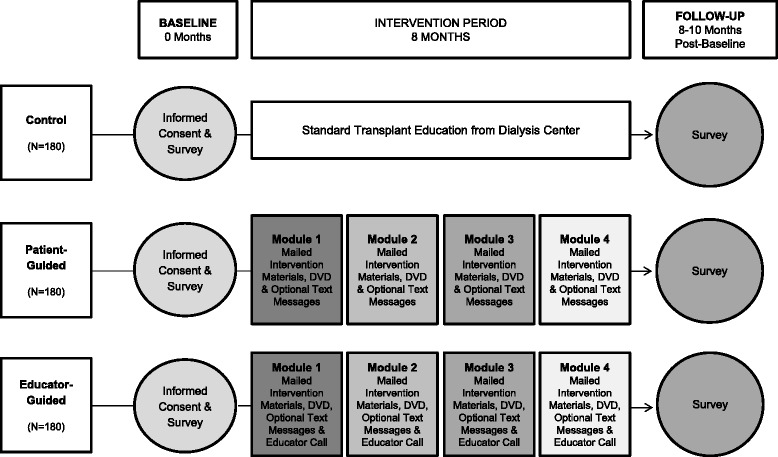
Explore Transplant at Home study design

This RCT has three conditions with equal allocation of patients to each condition: (1) the control condition or standard-of-care; (2) the *ETH* Patient-Guided (*ETH*-PG) condition; and (3) the *ETH* Educator-Guided (*ETH*-EG) condition (Fig. 2). All enrolled patients will complete a baseline survey and a follow-up survey, 8 to 10 months post-baseline. We will recruit 540 patients at the start of the 8 month educational intervention period to complete the baseline survey, with 180 patients in each condition. After attrition, 150 patients in each condition (*n* = 450 total) are expected to complete the follow-up survey.

### Patient Recruitment Eligibility, and Retention

The sample for our study will be drawn from the MoKP patient roster plus additional patients from Missouri dialysis centers. To enroll in the RCT each subject must: (1) be 18–74 years of age, (2) self-identify as Black or White race, (3) currently be on dialysis, (4) have a household income at or below 250 % of the federal poverty level, and (5) be able to speak and read in English. Participants who meet any of the following exclusion criteria are not enrolled: (1) has a visual and/or hearing impairment that would preclude him/her from watching and reading educational study materials, (2) has had a previous kidney transplant, and/or (3) has previously been told that they are not a candidate for transplant.

Those who are transplant-eligible will be contacted directly by phone and via mailed letters, inviting them to participate in our trial. Additionally, Kilgore’s Pharmacy, contracted by the University of Missouri Health System to support patients who receive medication assistance through MoKP, will insert flyers advertising the *ETH* research study into patients’ prescription medication refills. After being informed of the risks and benefits of the trial, patients will be asked to give verbal informed consent to participate. If a patient agrees to participate, he or she will be randomized to one of the three educational conditions using an unrestricted (simple) random allocation sequence implemented within the trial’s data capture software, after they complete the baseline survey. The randomization sequence was created by a data manager using a random sequence generator, is stored in an encrypted spreadsheet, and is not accessible by the principal investigator. This protocol has been approved by the University of California, Institutional Review Board (#14-000802), the University of Missouri, Columbia Institutional Review Board (#00048966), and is registered at ClinicalTrials.gov (#NCT02268682).

### Survey Timepoints

#### Baseline Survey

Patients will complete a short phone screening (5 min.) to assess whether they meet the eligibility criteria and if so, are invited to participate, review the informed consent sheet with a surveyor, and then complete a 45-minute baseline survey. The survey assesses demographic, clinical and cultural factors, socioeconomic transplant derailers, baseline level of transplant knowledge, decisional balance, self-efficacy, and any steps they may have taken to learn about staying on dialysis, DDKT or LDKT. Patients will receive $25 for completing the baseline survey.

#### Follow-up Survey

After the intervention period (approximately 8–10 months post-baseline) a 35-minute follow-up survey will be completed over the phone to assess changes in patients’ level of transplant knowledge, decisional balance, self-efficacy, informed decision-making, decisional conflict, and any steps they may have taken to learn about staying on dialysis, DDKT, or LDKT. They will also complete a process evaluation about the helpfulness of the *ETH* program. Patients who are deemed ineligible for transplant or receive a transplant during the interim period between the baseline and follow-up surveys will be given only an abbreviated set of questions during the follow-up survey that excludes any questions about their pursuit of transplant. Patients will receive $50 for completing the follow-up survey.

### Outcomes

#### Transplant Knowledge

Our primary outcome measure is level of DDKT and LDKT knowledge. Patients will be asked 17 true/false and 8 multiple choice questions about the basic facts, advantages, risks and outcomes of DDKT and LDKT to assess knowledge levels (e.g., “Transplant recipients are at risk of developing high blood pressure and high cholesterol,” “The transplant team will let a living donor back out from donating on the day of the surgery”).

#### Informed Decision Making

Informed Decision-Making will be assessed in two ways. First, patients will be asked three questions: “I have all the facts I need to make an informed decision about whether to pursue DDKT/LKDT/remain on dialysis” (agree/disagree). Each of these questions will be treated as an individual item and analyzed separately. We also will administer the decisional conflict scale [49] to assess factors contributing to patients’ uncertainty in making health-related decisions, and patients’ assessment of their perceived effective decision-making.

#### Small Steps toward DDKT and LDKT

Several action steps toward pursuit of both DDKT and LDKT will be assessed using validated measures (e.g., “Do you plan to call the transplant center to begin evaluation,” “Do you plan to share your interest in living donation with your family and friends?”) [50, 51], each of which the patient will report as having “already done,” “planning to do,” or “don’t plan to do.”

#### Decisional Balance and Self-Efficacy

Validated Decisional Balance measures will assess patients’ perceived importance of the possible positive and negative outcomes of LDKT and DDKT. Patients will be given the prompt “How important is this statement to your decision about transplant?” and then be asked to respond to 24 positive and negative statements (e.g., “I would not have to be on dialysis,” “I would feel guilty having someone donate to me”). Patients will be prompted to respond on a 5-point Likert-type scale (1 = "Not important" through 5 = "Extremely important") [50, 51]. A Self-Efficacy scale measures how confident an individual is in their ability to pursue transplant if they encounter challenges along the way with 14 questions. Patients are asked, “How confident are you that you could get a transplant even if…,” followed by a potential challenge, “You didn’t have your own transportation to get to the transplant center?” Patients responses will range from (1) = " Not at all confident" to (5) = " Completely confident" [50, 51].

### Predictors and Covariates

#### Demographics Clinical and Cultural Factors

Demographic clinical, and cultural characteristics will be assessed including age, sex, race/ethnicity, whether or not a patient is on dialysis and what type (i.e., hemodialysis or peritoneal), and patient comorbidities (e.g., polycystic kidney disease). Additionally, a patient’s health-related quality of life will be measured with the use of the Health Related Quality of Life-4 (HRQOL-4) scale [52] and medical mistrust will be measured using the Medical Mistrust Index, a validated scale that assesses how much patients trust health care organizations (e.g., “When healthcare organizations make mistakes they usually cover it up”) [53, 54].

#### Socioeconomic Transplant Derailers

We will also assess potential SES derailers that may influence patients’ experience with transplant evaluation including level of education, employment status (full time, part time, disability, other financial assistance programs, no employment), and the quality of health insurance they have (private, government, multiple sources, no insurance). Additional SES derailers assessed include feelings of safety in their neighborhood, family obligations, income vulnerability [55], and access to transportation [56]. We will also measure patients' access to multiple technologies and resources, such as having a computer or cell phone, access to the internet, and a DVD player.

#### Prior Transplant Education

As one of the primary outcome measures is transplant knowledge, patients’ level of prior LDKT and DDKT education will also be evaluated. Patients will be asked a series of four Yes/No questions about their past behaviors (e.g., “Have you read brochures about transplants?”), and if a patient positively endorses a statement, they will be asked how many hours they have dedicated to each educational activity.

#### Health Literacy

Patients will respond to two items: “How often do you have someone (like a family member friend, hospital/clinic worker or caregiver) help you read hospital materials?” and “How confident are you filling out forms by yourself?” [57].

#### Evaluation and Process Measures

In the follow-up survey, patients will be asked about the helpfulness of the *ETH* resources. Patients will also be asked whether they agree with 7 statements relating to how helpful they viewed the materials provided to them (e.g., “The materials were easy to understand,” “The materials were overwhelming”) and the educator conversations (e.g., “The Explore Transplant Educator was helpful”, “The Explore Transplant Educator listened to what I had to say”).

### Data Management and Statistical Considerations

#### Data Management

To ensure participant confidentiality and privacy all data will be stored in university-maintained, secured servers. All study data will be captured in electronic databases within the Research Electronic Data Capture (REDCap) system [58]. Study personnel can check on patients’ records by examining their data entry form or through reports generated in REDCap. The records of patients who refuse to participate or are never successfully recruited into the study will be retained in the REDCap registration database and de-identified at the end of the study so that patterns in recruitment can be analyzed and reported.

#### Power and Sample Size

Power analyses were based on changes in transplant knowledge, our primary study outcome. The study design and analyses were treated as a test of the differences in mean knowledge score change between patients in the *ETH*-PG, *ETH*-EG, and Control conditions, 8–10 months post-baseline. Power calculations were based on the number of dialysis centers per condition, the number of individuals per dialysis center, the expected intra-class correlation, and the estimated variability of the outcome variable [59]. Based on the original *ET* trial [19], we estimated that a mean knowledge change score of 2.0 points would be needed to detect a significant difference between patients receiving standard-of-care, *ETH*-PG, and *ETH*-EG. It was assumed that patients coming from the same dialysis centers would have correlated knowledge scores. Since we are examining 3 conditions, a Bonferroni correction was required to adjust the Type 1 α for comparison of 3 group means (0.05/3 = 0.017). Based on these assumptions, we calculated that a design of 150 patients per condition, 450 patients total, will be required to find a mean change of 2.0 points in knowledge between groups at 90 % power. Expecting an approximately 20 % attrition rate over time, we are oversampling (180 patients per condition) to ensure that we have sufficient power to be able to assess our primary endpoint at the completion of the study period.

#### Statistical Analyses

Where feasible and appropriate, multiple imputation will be used to account for missing data [60]. Multilevel random effects models (MRMs) will also be used to account for correlated data (dialysis center clustering and serial measurement). We will compare the characteristics of patients who refuse to join the study or are never successfully contacted to those who do not, as well as patients who drop-out to those who do not, to determine if the patient selection procedure has biased the sample.

We will compare the difference in mean knowledge change, change in self-efficacy, and change in decisional balance (baseline to follow-up survey) of participants in the 3 study arms using MRMs with normal outcome distributions. A MRM with a normal outcome distribution will also be used to test for mean differences in the Decisional Conflict Scale at 8 months post-baseline, and Rao-Scott χ^2^ tests adjusting for dialysis center clustering will be performed to test for differences in patients’ answers to the informed decision-making items on the follow-up survey: “I have all the facts I need to make an informed decision about whether to stay on dialysis/pursue DDKT/pursue LDKT” (agree/disagree). Rao-Scott χ^2^ tests will also be used to test differences in the proportion of patients in each condition who took each small step toward transplant (e.g., “Do you plan to share your interest in living donation with your family and friends?”), operationalized as the number who had not taken each step at baseline but had at follow-up.

The heterogeneity of treatment effect will be tested using interactions between the educational condition patients are assigned to and their demographic, psychosocial, and clinical characteristics (e.g., race, level of SES vulnerability, medical mistrust, health literacy), examining differences in changes in these groups' transplant knowledge, decisional balance, self-efficacy, small steps toward transplant, and informed decision-making using MRMs. These models will determine whether the *ETH* educational conditions are more or less effective for patients who: (1) have different SES barriers, (2) different levels of health literacy and medical mistrust, and (3) are Black or White. Finally, we will explore the impact of the text messages by comparing all study outcomes between patients who did and did not enroll in the texting program, stratified by whether the patient was randomized to the *ETH*-EG or *ETH*-PG condition, using MRMs.

## Discussion

Kidney transplantation has clear survival and quality-of-life benefits for patients; however, patients within low-income and minority populations continue to have limited access to the information they need to make an informed decision about their CKD treatment options. With the majority of dialysis patients dying within 5 years of starting dialysis [61], the importance of these patients receiving comprehensive education prior to their presentation at a transplant center about their other treatment options–DDKT and LDKT–cannot be understated. Since these patients are often less knowledgeable or ready to pursue transplant, transplant educational content must be simplified and made more culturally sensitive to honor patients where they are in their decision-making process about transplant.

Two recent transplant education interventions using print education, videos, and educators have been implemented with kidney patients prior to presenting to a transplant center in an effort to reduce disparities in pursuit or receipt of transplant [22, 62]. Compared to patients receiving usual transplant education in dialysis centers, a patient navigator intervention where previous kidney transplant recipients led dialysis patients through taking different transplant steps during a 2 year period (e.g., increasing interest in transplant, getting on the transplant wait-list, receiving a transplant) significantly increased the number of steps patients actually took, and was significantly associated with a higher likelihood of wait-listing. A second trial examined the impact of an educational intervention on transplant pursuit for CKD 3–5 patients recruited from community nephrology practices [22]. Compared to standard-of-care education, patients who received additional print and video education with support from a social worker were more likely to have discussions about transplant and take other LDKT steps (e.g., identify potential living donors). Also, the patients who only received educational materials were more likely to begin and complete transplant evaluation than the other two groups [22].

These studies show support for outreach-based, culturally-compentent educational approaches to be studied and further expanded in the *ETH* RCT described here. However, neither of these studies examined whether their respective interventions were effective when targeted toward low-SES patients. Since these patients may take more time to educate, we do not know whether it is feasible to overcome the greater level of challenges faced by dialysis providers in educating them about transplant.

Upon completion of this investigation, we will have assessed the effectiveness of a program that delivers health education directly to patients’ homes in small, digestible increments. Additionally, we will be able to explore the effectiveness of text messaging as a means of delivering health education to patients within a low-income population. Hopefully, these methods prove to be effective at alleviating staff burden within dialysis centers, educating patients in an engaging fashion, and providing a potentially cost-effective strategy for disseminating transplant education. Furthermore, through this trial, we will have developed an education program that could be delivered directly to patients through a healthcare organization, health insurance company, or other community partners—all organizations that have continued access to patients as a result of the managed care they provide. Next steps will include examining the effectiveness of *ETH* educational approaches with Hispanics, the most rapidly increasing portion of the ESRD population in the United States [1] who may face particular challenges around pursuing transplant [63, 64] and assessing the generalizability of these findings in other regions of the country.

## Abbreviations

ESRD: End-stage renal disease
CKD: Chronic Kidney Disease
LDKT: Living donor kidney transplant
DDKT: Deceased donor kidney transplant
ET: Explore Transplant
ETH: Explore Transplant at Home
ETH-PG: Explore Transplant at Home Patient-Guided Condition
ETH-EG: Explore Transplant at Home Educator-Guided Condition
TTM: Transtheoretical Model of Behavioral Change
RCT: Randomized controlled trial
SES: Socioeconomic status
MoKP: Missouri Kidney Program
REDCap: Research Electronic Data Capture
MRM: Multilevel Random Effects Model
